# A Simple and Safe Method to Create a Drainage Hole for Thick Skin Grafts

**Published:** 2017-10-04

**Authors:** Naohiro Ishii, Shigeki Sakai, Kazuo Kishi

**Affiliations:** Department of Plastic and Reconstructive Surgery, Keio University, Tokyo, Japan

**Keywords:** skin graft, hematoma, drainage hole, drainage, full-thickness skin graft

## DESCRIPTION

We developed a novel technique to create drainage holes for skin grafts simply and safely using a beaker, a rubber band, and gauze, which are easily obtained in the operating room.

## QUESTIONS

1. What data have been reported on the prevention of hematoma development in skin grafts?
2. What problems exist in the conventional method of creating a drainage hole in skin grafts?
3. What is the technique presented here for the creation of a drainage hole?
4. What are the merits of the present technique?

## DISCUSSION

Skin grafts can be partially or completely unsuccessful for various reasons. The most common reason for graft failure is hematoma formation between the skin graft and the wound bed, obstructing graft nutrition and revascularization.^1^^-^5 Many techniques for hematoma prevention have been reported in the literature, including tie-over dressing, negative pressure dressing, and careful hemostasis of the recipient site.^2^^,^^3^^,^^6^^-^8 In addition, the appropriate drainage of blood under the skin graft is also important, and a meshed skin graft is the typical technique to achieve this. However, other drainage techniques have not been reported in detail.

**Figure 1:**
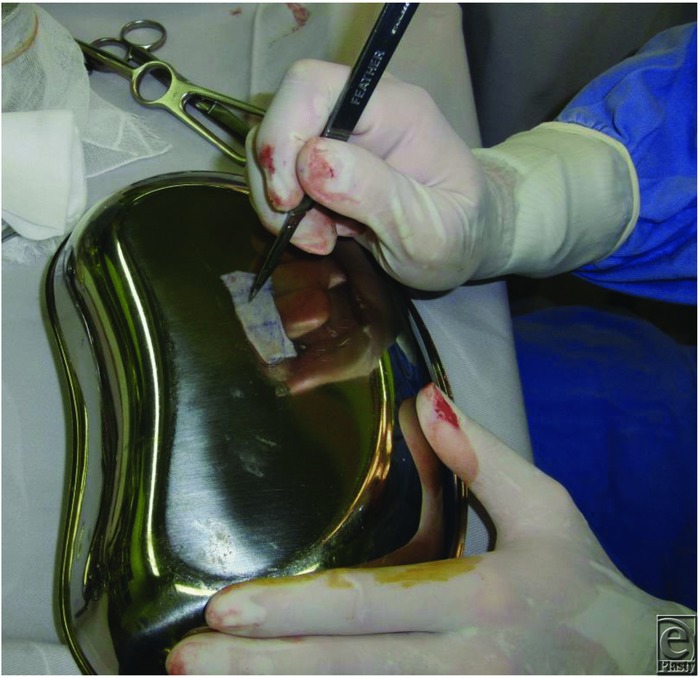
Creating drainage holes in a full-thickness skin graft using a metal plate as a desk pad.

**Figure 2:**
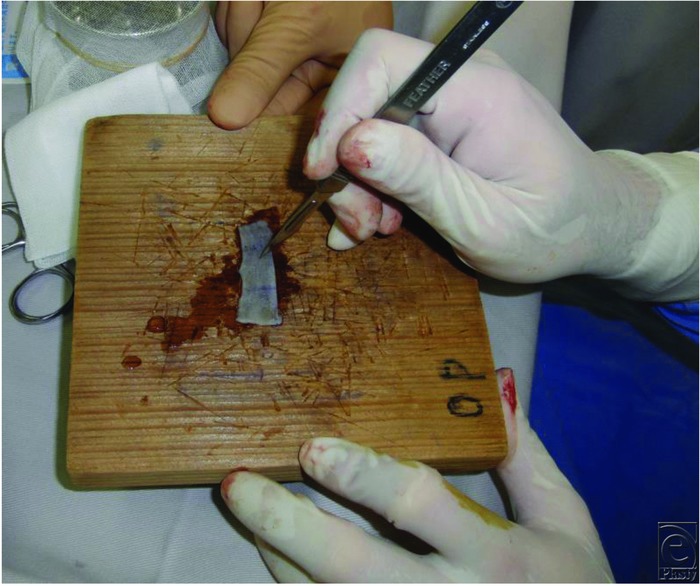
Creating drainage holes in a full-thickness skin graft using a wooden plate as a desk pad.

A surgical knife and a needle may be used conventionally to create a drainage hole in a skin graft in cases in which meshing is not appropriate due to cosmetic reasons. This method is often applied to full-thickness skin grafts using a metal or wooden plate as a desk pad (Figs 1 and 2). However, it is difficult to create a drainage hole effectively in a thick skin graft lying on a hard plate. Trauma to the plate should also be considered. Certain drainage holes can be created while floating the skin graft by hand; however, this method is not safe and cannot be performed by a single surgeon. Creating drainage holes after graft fixation carries the risk of trauma to the recipient site.

**Figure 4:**
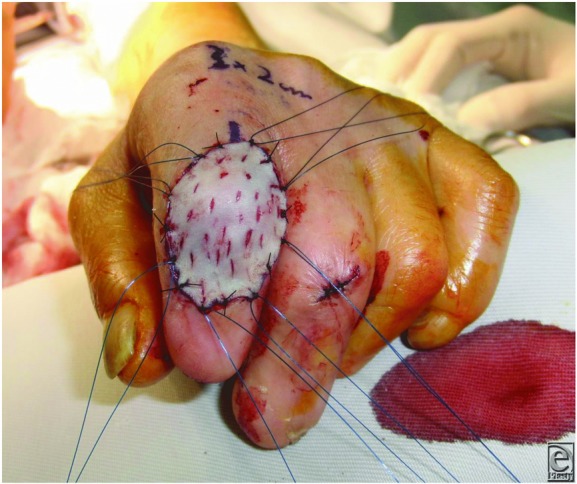

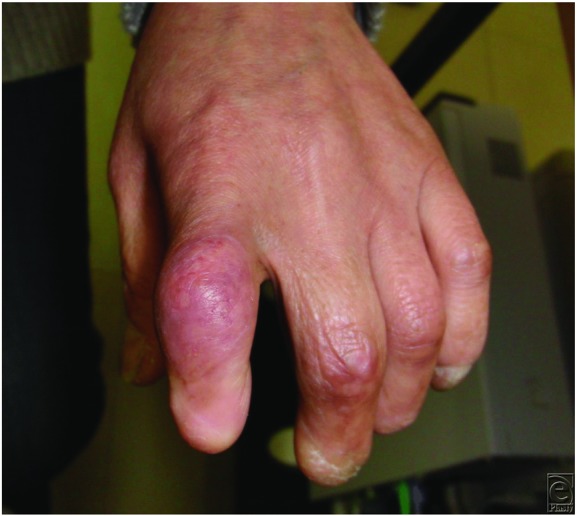
(a) A full-thickness skin graft using the present technique to address a skin defect of the dorsal finger. (b) Postoperative month 6. The skin graft was fully incorporated without hematoma.

The technique presented here for creating a drainage hole is shown in Figure 3 (video). The instruments necessary for performing this technique include a beaker, a rubber band, and gauze. First, the beaker is covered with a bifolded gauze that is fixed in a tense position using a rubber band. Then, the skin graft is spread out and placed on the gauze, and drainage holes are created with a surgical knife or needle going through the gauze. The skin graft on the gauze is shifted according to the required number of drainage holes. The skin graft is then washed with saline and grafted to the recipient site. The clinical application of this technique is shown in Figure 4.

The present technique has various merits. First, it is simple and safe, and a single surgeon can create the drainage holes certainly involving all layers of the skin graft. Second, the instruments necessary to perform this technique are used daily in the operating room; therefore, it is cost-effective. Even if the gauze is worn with many holes, it can be rapidly exchanged with a new gauze. In addition, even if the skin graft is large, drainage holes can be created evenly and wholly by shifting the graft, and drainage holes can be created more easily by creating tension in the skin graft at the margin of the beaker with the surgeon's finger.

We have applied the present technique in our daily practice for graft operations and consider it useful for simple and safe creation of drainage holes, particularly for thick skin grafts.

## Figures and Tables

**Figure 3:** Creating drainage holes in a full-thickness skin graft using the present technique.
